# Neural Synchronization from the Perspective of Non-linear Dynamics

**DOI:** 10.3389/fncom.2017.00098

**Published:** 2017-10-26

**Authors:** Ramon Guevara Erra, Jose L. Perez Velazquez, Michael Rosenblum

**Affiliations:** ^1^Laboratoire Psychologie de la Perception, Centre National de la Recherche Scientifique and Université Paris Descartes, Sorbonne Paris Cité, Paris, France; ^2^Neuroscience and Mental Health Programme and Division of Neurology, Department of Paediatrics, The Ronin Institute, Institute of Medical Science, Hospital for Sick Children, University of Toronto, Toronto, ON, Canada; ^3^Department of Physics and Astronomy, University of Potsdam, Potsdam, Germany

**Keywords:** brain synchronization, non-linear dynamics, neural synchonization, brain rhythms, epilepsy

The discovery of oscillations in brain activity is as old as electroencephalography (EEG), but only with the development of powerful imaging and computational techniques in the last decades it has been possible to start understanding the role of brain rhythms in brain physiology, pathology, and cognition. For this reason, the study of brain rhythms and synchronization of oscillatory activity is currently one of the hottest topics in neuroscience. Emerging from this research burst is the following: brain rhythms coexist with the background, non-oscillatory (noisy) neuronal activity, they appear to exist only at specific frequencies and they are generated in the brain as the coordinated output of large neuronal networks. In other words, under certain circumstances the normally very high-dimensional dynamics of neuronal networks activity collapses into low dimensional oscillatory modes. As evidenced by seizures in epilepsies and tremor activity in Parkinson disease, very rhythmic activity and excessive synchronization may be pathological for the nervous system (especially for the higher brain centers, as it may be adequate for other regions involved in the control of, say, breathing patterns). On the other hand, a large corpus of evidence has shown the connection of brain rhythms and neural synchronization to cognition. Experimental research on brain oscillations and neural synchronization has been accompanied by an intense effort to understand the generation of rhythms and the mechanisms of neural synchronization through computational modeling and non-linear dynamics. However, the two sides of this enterprise often lack a common language, methodology, and concepts. In this opinion article, we consider neural synchronization from the perspective of oscillation theory. We also describe recent experiments on epileptogenic activity in rat models, providing further evidence of the applicability of oscillator theory to a neuroscience context. Finally, we briefly compare the terminology used in physics and neuroscience in the context of synchronization phenomena.

A mathematical description of neural synchronization (Buzsaki, 2006; Nowotny et al., 2008) can be done within the framework of non-linear dynamics (specifically oscillator theory) that accounts for synchronization phenomena in general (Kuramoto, 2003; Pikovsky et al., 2003; Guckenheimer and Holmes, 2013; Landa, 2013). Central to synchronization theory is the concept of self-sustained oscillator: an autonomous continuous-time dynamical system with a special kind of attractor, a limit cycle (Guckenheimer and Holmes, 2013; Strogatz, 2014). Due to the existence of the limit cycle, a stationary self-sustained oscillation can be described in terms of a phase, which is a variable parameterizing the motion along the cycle. In general, when oscillators interact they adjust their phases and frequencies and can eventually synchronize (Kuramoto, 2003; Pikovsky et al., 2003; Landa, 2013). Synchronization manifests itself via phase and frequency locking (entrainment) (Pikovsky et al., 2003). When coupling is weak the amplitudes are nearly unchanged and the dynamics admits a reduced description in terms of phase models (Kuramoto, 2003; Pikovsky et al., 2003). It is worth emphasizing that synchronization applies to self-sustained oscillators only, but their presence in the nervous system is not *a priori* clear (Perez Velazquez, 2005). Thus, the applicability of synchronization theory to a specific neuroscientific context should be analyzed in a case-by-case fashion.

Although the limit cycle oscillation is an idealization, not typically fulfilled in real systems, this concept can be extended to noisy and/or weakly chaotic systems for which the phase can be defined. This is crucial for applications in neuroscience because these kind of systems can gives rise to a narrow band signal as the one observed in electrophysiological recordings of the brain (Pikovsky et al., 2003).

Oscillatory activity in the nervous system appears at different spatio-temporal scales, and they include spike trains, local field potentials, and large-scale oscillations (Varela et al., 2001). Quite often, individual neurons exhibiting rhythmic spiking or bursting activity can be considered as self-sustained oscillators (Buzsaki, 2006; Ermentrout and Terman, 2010). The existence of macroscopic self-sustained oscillators is perhaps more controversial. It has been for long hypothesized that macroscopic rhythms like pacemaker activity, motor pattern generation and cortical (alpha, theta, gamma) rhythms emerge through a Hopf-like bifurcation. In this case, the corresponding neuronal population can be considered as a self-sustained oscillator (noisy periodic or chaotic). Computational models and *in vitro* experiments point in this direction at least in the important case of the gamma rhythm (Akam et al., 2012; Kotani et al., 2014). Here, we emphasize the possibility that macroscopic self-sustained oscillators can be experimentally studied in the nervous system. Thus, we have recently found *in vivo* experimental evidence for the existence of a macroscopic self-sustained oscillator in the brain of epileptic rats (Perez Velazquez et al., 2015). The approach used was to study the response of thalamocortical circuits to periodic pulse stimulation, in a rat model of absent seizures. During these absent seizure, brain activity is rhythmical (spike and waves discharges, that were recorded with intracranial electrodes in cortical regions), and it was observed that stimulation with a periodic pulse (in the thalamus) changed the frequency of the rhythm. It was found that the thalamocortical network (which is crucial for the generation of seizures) exhibited the phenomenon of frequency locking, at least within a restricted frequency range. The characteristic Arnold tongues were experimentally observed. In conclusion, this activity appears to represent a macroscopic self-sustained oscillation.

We propose that other macroscopic rhythms in the brain could be investigated using analogous techniques. Indeed, a similar approach was recently implemented on human subjects to investigate the functional relevance of brain oscillations in the alpha frequency range (8–13 Hz), via rhythmic light stimulation (Notbohm et al., 2016). We emphasize that these results (Perez Velazquez et al., 2015; Notbohm et al., 2016) are also valid for a noisy limit cycle or a weakly chaotic oscillator, as explained above.

A related and very important methodology in the research of rhythms is the phase response—or resetting—curve (PRC) (Schultheiss et al., 2011). It measures the phase perturbation of an oscillator as a function of the phase of the oscillator at the moment of stimulation. It allows experimentalists to study the properties of the underlying oscillators, generating the rhythm. As an example of its applicability, in a study on the same absent seizure model described above, the dynamics of thalamic and cortical networks was found to be compatible with a very simple system of coupled oscillators. The thalamus and the cortex were described as two oscillators with interactions given by the experimentally obtained PRCs (Velazquez et al., 2007). Taken together, these works (Velazquez et al., 2007; Perez Velazquez et al., 2015) demonstrate that oscillator theory fairly describes several aspects of the inherent dynamics of seizures in the thalamocortical system. This has important implications for seizure control, given that such simple models can be studied using non-linear dynamics both theoretically and in simulations. For example, such models can be used for seizure cancelation in medical settings, through the implementation of feedback control systems that could induce desynchronization of the thalamocortical network as it was recently achieved in other *in vivo* seizure models using a feedback, or closed-loop, protocol (Salam et al., 2015).

The term oscillatory synchrony, neural synchrony, or neural synchronization is widely used in the neuroscience literature referring to somehow related but slightly different concepts, depending on the context (Varela et al., 2001). In its most common connotation, the term is associated to temporal correlation between brain signals. This is an operational definition, and a statistical one—based on how synchrony between brain signals is measured. For this reason, this definition is scale-dependent. At the cellular level, it is measured by cross-correlograms of spike trains, typically correlating with local field potential oscillations (Friston, 1997). In other words, it is understood that neurons synchronize if they spike simultaneously. On the other hand, the physical definition of synchronization is more general, allowing for the possibility of phase shift between synchronized signals. At the cortical column level, the postsynaptic activity of networks of pyramidal cells is synchronized, giving rise to a measurable electrophysiological signal [such as electro and magnetoencephalographic (EEG/MEG) signals]. The power of these signals is a measure of correlated activity in the underlying networks. By construction, both cross-correlograms and cortical electrophysiological signals measure both phase and amplitude correlation in neuronal activity. Synchrony can also be measured between spatially separated brain regions—the so-called large-scale neural synchronization—representing in principle phase synchronization (measuring phase correlation but not amplitude correlation). However it should be noticed that large-scale neural synchrony is not a direct measure of coupling between neuronal sources due to the fact that scalp-measured signals are a signal superposition of brain sources (Nunez et al., 1997; Nolte et al., 2004; Meinecke et al., 2005).

Several measures of interdependence of neural signal synchrony and of their synchrony in particular exist in the literature, based on time series analysis techniques (see Kreuz, 2011 and Lehnertz, 2011; and references therein). These measures capture both linear (spectral coherence and cross-correlation) and non-linear (Granger causality, phase synchronization, non-linear interdependences mutual information, and transfer entropy) aspects of interactions. Measures of synchronization such as mean phase coherence and phase locking values has been specifically introduced within a neurophysiological context (Lachaux et al., 1999; Mormann et al., 2000). Most of the measures used to detect neural synchrony are based on pairwise analysis of bivariate signals. However, in a typical experimental setting there are many channels and a bivariate analysis does not account for the full covariance information of a multichannel set. For this reason, genuine multivariate analysis has been introduced to analyze neurophysiological signals (see Pereda et al., 2005 and references therein).

Characterizing interactions between oscillatory systems from observations is a hard inverse problem even for noise-free and stationary physical systems (Kralemann et al., 2007). In fact, information about the directional coupling cannot be derived from synchrony measures and requires a reconstruction of the dynamical model. A particular problem, not very well known in neuroscience community is that phases extracted from the data, e.g., with the help of the Hilbert transform, differ from the phases of the underlying dynamical systems. The corresponding dynamical systems, reconstructed from data, are not invariant, in the sense that they depend on the measured signals as well as on the embedding method used for the construction of phases. This problem can be partially solved by means of a special transformation (Kralemann et al., 2007, 2008, 2011). We emphasize that this problem is not equivalent to the volume conduction problem, because it appears whenever scalar observables are measured (the underlying vector of state variables is unknown), which is the case for most experimental conditions. Indeed, the problem should persist even in the absence of superposition of signals, as, for example, in intracranial EEG recordings.

Synchronization has been associated in certain contexts to the classical definition used in non-linear dynamics, while keeping intact its different neural connotations. For example, an influential book in mathematical neuroscience (Izhikevich, 2007) states that “partial synchrony in cortical networks is believed to generate various brain oscillations, such as the alpha and gamma EEG rhythms. Increased synchrony may result in pathological types of activity, such as epilepsy” and also that “There is an ongoing debate on the role of synchrony in neural computation…devoted to the binding problem.” Also in the mathematical neuroscience field, synchrony has been related to brain rhythms such as those generated by central pattern generators (Ermentrout and Terman, 2010).

One problem with this plurality of interpretations due to field-specific definitions is the risk of extrapolating methods and results from one field to another, without careful verification of the underlying assumptions and the limits of applicability of oscillation theory. Indeed, as noted above, neural synchrony derived on the basis of electrophysiological recordings is not necessarily a measure of phase synchronization alone (as it may be amplitude-dependent) and it is not a direct measure of neural coupling (due to the signal superposition/volume conduction problem and the non-invariance of experimentally determined phases). Furthermore, the concept of coupling itself may have different meanings in different fields: in physics coupling is typically understood as an existing physical connection between systems (e.g., neurons are connected via synapses), while in neuroscience quite often coupling between signals is understood as a measure of correlation (synchronization). In this regard, several notions of “connectivity” have emerged in neuroscience, including structural and effective connectivity.

In the cognitive neuroscience literature, entrainment is typically restricted to phase-locking, and not frequency-locking [for example, “oscillations in the sensory cortices will entrain (phase-lock) to the events in the attended stream” (Lakatos et al., 2005) or “entrainment of low-frequency oscillations involves a reorganization of phase so that the optimal, in this case most excitable, phase comes into line with temporally expected critical events in the ongoing stimulus” (Henry and Obleser, 2012)]. We emphasize here that without a self-sustained oscillator, one cannot talk of entrainment, as it is understood in physics. In particular, for a self-sustained oscillator, oscillations should persist after the external stimulation is over; we remark that self-sustained oscillations in physical language are equivalent to “endogenous neural oscillations” in neuroscience. They constitute rhythmic fluctuations in the excitation-inhibition cycle of neuronal populations (Bishop, 1932; Buzsáki and Draguhn, 2004; Lakatos et al., 2005). Furthermore, entrainment can be easily confused with resonance: the response of a passive system to external driving. In terms of dynamical systems, the distinction between both systems resides in the existence of a zero Lyapunov exponent for self-sustained oscillators, and its absence in driven oscillators (Pikovsky et al., 2003). In other words, an autonomous self-sustained oscillator has a neutrally stable phase that can be adjusted by the external force, whereas in driven systems the phase of oscillations is unambiguously related to the phase of the external force, so it cannot be easily shifted and therefore it cannot be synchronized. However, in experimental settings, a system that is being driven could mimic entrainment (Pikovsky et al., 2003). As an example, consider the important line of research devoted to the study of entrainment of oscillatory brain activity to stimuli in the auditory cortex (Lakatos et al., 2005, 2008; Schroeder and Lakatos, 2009). In this case, are we really talking about entrainment or resonance? We propose that this should be experimentally assessed in a case-by-case fashion: by just observing the adjustment of an endogenous rhythm to the external stimulation, without further investigation, it is difficult to distinguish between entrainment and resonance. However, indirect evidence can also help establishing the distinction. In this particular case, there is physiological evidence for synchronization phenomena in early stages of auditory perception. Indeed, the mechanical properties of the basilar membrane's response becomes highly non-linear near the resonance, probably because the system operates close to a critical point, a Hopf bifurcation (the birth of a limit cycle) (Ospeck et al., 2001; Hudspeth et al., 2010). A Hopf instability could also help understanding the cochlea tuning curve (Magnasco, 2003). Given that the mechanical and neural responses of the cochlea are strongly connected, neural self-sustained oscillators could indeed exist at early stages in auditory processing.

## Author contributions

RG wrote the original version and revised the manuscript and contributed to its discussion, JP reviewed and made changes to the manuscript and contributed to its discussion; MR wrote parts of the manuscript and contributed to its discussion.

## Conflict of interest statement

The authors declare that the research was conducted in the absence of any commercial or financial relationships that could be construed as a potential conflict of interest.
